# Editorial: Transcription regulation — Brain development and homeostasis — A finely tuned and orchestrated scenario in physiology and pathology, volume II

**DOI:** 10.3389/fnmol.2023.1280573

**Published:** 2023-09-05

**Authors:** Estela M. Muñoz, Verónica Martínez Cerdeño

**Affiliations:** ^1^Institute of Histology and Embryology of Mendoza (IHEM), National University of Cuyo (UNCuyo), National Scientific and Technical Research Council (CONICET), Mendoza, Argentina; ^2^Department of Pathology and Laboratory Medicine, Institute for Pediatric Regenerative Medicine, Shriners Hospitals for Children of Northern California, and MIND Institute at the UC Davis Medical Center, University of California Davis School of Medicine, Sacramento, CA, United States

**Keywords:** transcription, transcription factors, brain, neurodevelopment, diseases

The Research Topic (RT) discussed herein is the second volume of a Research Topic focused on transcription factors (TF) and transcription regulation in the healthy and diseased brain. The previously published first volume was compiled into a well-visited and well-read E-Book (with more than 61,000 total views and downloads, to date) (Muñoz et al., 2021, 2022). Encompassed in this second volume are eleven peer-reviewed manuscripts including five original articles, four reviews, one mini review, and one brief research report. Seventy-five authors took part in this initiative, from eleven countries: Argentina, Australia, Austria, Denmark, Germany, Italy, India, Spain, Sweden, United Kingdom, and United States.

Among the interesting contributions, Xia et al. summarize the current knowledge of transcription regulation of the respiratory neurons in the brainstem. These neurons are responsible for generating, monitoring, and adjusting breathing patterns in response to external and internal demands. Thus, these motor neurons represent key actors of breathing (or respiration), which is an elementary but complex and dynamic behavior. First, the authors present generalities of the transcriptional programs that drive the anterior-posterior and the dorsal-ventral patterning of the developing brainstem, emphasizing its neuronal diversity. Next, they discuss the transcriptional control that secures the specification of certain groups of neurons that form the central respiratory system in the hindbrain. This discussion includes the pontine groups, and the neurons of both the dorsal and the ventral medullary respiratory columns. Lastly, the authors consider disturbances of TF-coding genes (e.g., mutations in *PHOX2B* and *LBX1*) that cause congenital respiratory syndromes, as a possible path to further understand the genesis, specification, and function of respiratory neurons and the basis of breathing. The review is well-illustrated, and the readers can visit the following references for further details (Alexander et al., 2009; Hernandez-Miranda and Birchmeier, 2015; Hernandez-Miranda et al., 2017, 2018; Isik and Hernandez-Miranda, 2022; Lowenstein et al., 2023).

The prefrontal cortex (PFC) is a large brain region that compromises a diversity of neuronal and non-neuronal cell types. The timely appearance and connectivity of these cells during development are crucial for normal PFC anatomy and functions (Cadwell et al., 2019). PFC is involved in fundamentally important brain processes, such as cognition and memory. In this RT, Singh and Tiwari focus their brief research report on the developing human PFC. The authors aimed to identify transient cell states that emerge during the cellular developmental trajectories of the major cortical cell types and their underlying gene regulatory circuitry, with respect to early (8–13 gestational weeks, GW), mid (16–19 GW), and late (23–26 GW) fetal stages. The authors reanalyzed two publicly available single-cell RNA sequencing (scRNA-seq) datasets (Zhong et al., 2018; Bhaduri et al., 2021), by using advanced computational approaches including CellRank for cellular trajectory reconstruction (Lange et al., 2022), the partition-based graph abstraction (PAGA) method for cell fate connectivity (Wolf et al., 2019), and the iQcell platform for gene regulatory network dissection (Heydari et al., 2022). The authors present and discuss cellular trajectories for neuronal and oligodendroglial lineages and key drivers of cell fate decisions, as well as novel TF regulatory networks [e.g., SOX8/SOX10-driven gene expression programs for the oligodendrocyte progenitor cell (OPC) lineage (Stolt et al., 2005; Garcia-Leon et al., 2018)]. They concluded that the precise characterization of the cellular and molecular heterogeneity of the developing and mature human cortex is essential to understanding brain functions and regulation, and to identifying the causes and potential interventions for neurodevelopmental and neurological disorders.

Cell state transitions and the underlying transcriptional regulatory mechanisms during the neural induction stage were studied *in vitro* by Gupta et al.. They performed a longitudinal analysis of the transcriptome of human induced pluripotent stem cells (hiPSC), as these cells were undergoing 8-day neural induction (Shi et al., 2012; Lee et al., 2020). The authors identified distinct functional modules by following the temporal dynamics of key TFs (e.g., OTX2, KEAP and NRF2), and subsequent changes in the expression profiles of their target genes. These modules are related to the transition whereby pluripotency is lost as distinct neural identity is gained, cell cycle progression, metabolic reprogramming, stress response, and genome integrity. Some of the modules may operate throughout the entire initiating process, but they may use different gene signatures. Finally, the authors focused their attention on the precociously expressed TF OTX2 (Rath et al., 2006; Acampora et al., 2013), for which they propose diverse mechanisms throughout the neural induction stage. This was validated by knocking down *OTX2* expression by CRISPRi prior to neural induction. The authors emphasize the widespread remodeling of the whole cell that takes place during the induction of one specific lineage.

The hiPSC model was also used by Schuster et al. to study GABAergic interneuron development and function in the Mowat-Wilson syndrome (MWS). This epileptic neurodevelopmental disorder is caused by heterozygous variants in the *ZEB2* gene, which encodes the TF ZEB2 (Mowat et al., 1998; Zweier et al., 2002). Inhibitory cortical and hippocampal GABAergic neurons require a functional ZEB2 for correct migration and differentiation, to maintain hence a balanced overall neuronal activity (Miquelajauregui et al., 2007; Van Den Berghe et al., 2013). In this original article, the authors confirm that ZEB2 haploinsufficiency alters the GABAergic fate trajectory and its function. They transcriptomically compared hiPSC lines derived from fibroblasts of two related MWS subjects carrying the heterozygous non-sense variant c.1027C>T (p.Arg343^*^) in exon 8, with those from two healthy donors. The authors used a 65-day protocol that started with neural induction and concluded with GABAergic differentiation (Schuster et al., 2019). Dysregulation of specific genes related to transcription control, cell fate decisions and patterning, and epilepsy, is presented and discussed. Mixed cell identities to the detriment of the GABAergic neuron type, impaired migration of neural stem cells (NSC), and altered electrophysiological properties of differentiated GABAergic interneurons, were found. This finding has led the authors to propose their data (and the *in vitro* MWS model) as a framework for further study to better understand the underlying cellular and molecular basis behind the neuropathogenesis and seizures in MWS, that could potentially lead to interventions of the disease.

Arcuschin et al. contributed to this Research Topic with a mini review that links mechanisms of robustness with neurodevelopment. The robustness of a biological system is defined as its ability to buffer internal and external perturbations, generated by genetic and epigenetic variations, molecular noises or environmental fluctuations, in favor of promoting a reliable output or a particular phenotype (Barkai and Shilo, 2007; Felix and Wagner, 2008). Neurodevelopment in multicellular organisms involves complex signaling interactions between participating cells, as they develop (Silbereis et al., 2016). Fluctuations in these signals may impact gene regulatory networks (GRN) that control cell type trajectories and patterning, as well as other aspects not discussed in this article. Therefore, robustness strategies are essential during normal neural development. First, the authors compare the concept of robustness with the lack of phenotype variability. Next, they present the developing nervous system, with its cellular heterogeneity, sequential cell type genesis (neurogenesis followed by gliogenesis), and broad spectrum of internal and external regulatory signals [e.g., gradients of morphogens such as Shh (Sagner and Briscoe, 2017)], as a model to study and define mechanisms of robustness. At the transcriptional level, the authors address the redundancy of TFs due to gene duplication [e.g., Gsx1 and Gsx2 (Chapman et al., 2018)], as well as the presence of multiple TF binding sites within a specific enhancer and multiple enhancers for the expression of a particular gene (e.g., *Shh, Krox20*, and *Pax3*), as mechanisms to buffer perturbations during neurodevelopment. In addition, they present miRNAs [e.g., miR-9a for the Senseless TF (Cassidy et al., 2013)], and the chromatin conformation and promoter architecture, as having roles that contribute to gene expression robustness. At a higher and more complex level (from cells to systems), the authors consider the robustness of interlocking GRNs, as well as the influence of cell-to-cell interactions. Finally, the authors discuss how alterations in robustness mechanisms, or their surpassing by excessive disruptors, may lead to neurodevelopmental disorders including microcephaly, FXS (fragile X syndrome), and ASD (autism spectrum disorder).

The potential association of FXTAS with variations in the human *AQP4* gene was studied by Elias-Mas et al.. Aquaporin 4 (AQP4) is a highly expressed brain water channel that is essential for fluid homeostasis (Szczygielski et al., 2021). In fact, the dysfunction of AQP4 has been related to several degenerative conditions including Alzheimer's (AD) disease and Parkinson's (PD) disease (Mader and Brimberg, 2019). FXTAS (fragile X-associated tremor/ataxia syndrome) is also a neurogenerative disease with a late onset and impaired motor and cognitive functions. FXTAS is linked to *FMR1* gene premutations (55–200 CGGs) (Cabal-Herrera et al., 2020). Not all *FMR1* premutation carriers, however, present clinical symptoms. An investigative search to identify additional risk factors led the authors to conduct this study. Elias-Mas et al. compared the frequency of seven disease-related single nucleotide polymorphisms (SNP) across the *AQP4* gene in both FXTAS and non-FXTAS individuals, by genotyping 160 *FMR1* premutation Caucasian carriers (59% were clinically positive for FXTAS). No significant association with the risk of developing FXTAS was found for any of the analyzed SNPs neither for the major and minor allele haplotypes (HTMa and HTMi); conclusion that was maintained after correction by multiple tests. The authors discuss the limitations of their study regarding sample sizes and age differences between FXTAS and non-FXTAS groups. They also stated the need to further study AQPs and the glymphatic system, to either confirm or discard their involvement in FXTAS initiation and its progression.

Regarding genetic defects and developmental abnormalities, Bernardinelli et al. present two novel sequence variants of *POU3F4*: g.5284delA and g.6045T>A, which encode truncated protein products of the TF POU3F4, p.S74Afs^*^8 and p.C327^*^, respectively. These mutations were identified by genotyping two male Caucasian subjects, who had been diagnosed with typical hallmarks of POU3F4-related hearing loss [incomplete partition of the cochlea type 3 and enlarged vestibular aqueduct (Roesch et al., 2021)]. The authors studied the pathogenicity of these novel sequences in cell-based assays, by analyzing their subcellular distribution and transcriptional activity. Of these defective proteins, only p.C327^*^ did successfully reach the cell nucleus, which was explained by the presence of two of the three nuclear localization signals (NLS) predicted in the wild type (WT) POU3F4. However, it was noted that the spatial pattern of the nuclear p.C327^*^ was different than that of the WT POU3F4. In addition, both variants failed to properly activate the expression of a reporter gene and to enhance the transcription of POU3F4-driven genes, which were identified by RNA-seq under the influence of the WT TF. Among these genes, the authors selected *SLC6A20* for further analysis due to the essential function of SLC6A20 as an amino acid transporter, as well as the association that this class of transporters has with some pathologies, notably hearing loss and cognitive impairment (Swarna et al., 2004; Takanaga et al., 2005; Broer et al., 2008). However, a straightforward correlation between genotypes and phenotypes was not found in these individuals, suggesting that a higher level of complexity may be involved.

Kaltschmidt et al. address the human genetics of NF-kappa B signaling, as it applies to neurodegenerative diseases and malignant brain tumors. Although the NF-κB family of TFs has been extensively involved in inflammation and cancer, it is also well-accepted that it influences a broad range of cellular processes, including within the nervous system (Kaltschmidt and Kaltschmidt, 2009). NF-κB functions are executed via a selection of canonical, non-canonical and atypical pathways (Kaltschmidt et al., 2021), all of which are concisely presented in this article. Then, the authors discuss the synaptic location of NF-κB in certain brain regions (e.g., cerebellum, cortex, and hippocampus), and its retrograde transport from the synapsis to the cell nucleus. The authors suggest that these characteristics might make NF-κB a suitable messenger to communicate feedback for regulating gene expression, which could contribute to synaptic plasticity (Kandel, 2001). Additional related topics are introduced, including a comparison of constitutive vs. inducible NF-κB activities in glutamatergic neurons, and animal models to study the impact of altered NF-κB signaling in learning and memory, and in brain regeneration. In their discussion of the pathophysiology and genetics of both AD and PD, and how they are impacted by the TNFα/NF-κB pathway, the authors propose new target genes, as well as new theories and hypotheses (Snow and Albensi, 2016; Panicker et al., 2021; Bellenguez et al., 2022). Finally, the authors summarize current knowledge related to NF-κB signaling and glioblastoma multiforme (GBM), in terms of growth, invasiveness, and angiogenesis (Smith et al., 2008; Wang et al., 2021). The authors conclude that the recent genetic evidence around the NF-κB pathway may lead to new research and therapeutic approaches for treating neurodegenerative diseases and brain tumors.

Maurya et al. contributed to this RT with an extensive review about microglia, and their involvement in brain development, homeostasis, diseases, and therapeutics. The authors discuss the origin of these innate immune cells (Sierra et al., 2016), and the mechanisms of their interaction with both neurons and non-neuronal cells, including those mediated by exosomes (Muñoz, 2018, 2022; Kalluri and Lebleu, 2020; Guo et al., 2021; Hazrati et al., 2022). Key genes in microglia biology are discussed, as well as their regulation and dysregulation, especially related to several pathological conditions [e.g., AD, PD, multiple sclerosis (MS) and neurodevelopmental diseases]. Included among these genes, are those that encode the TFs PU.1, Sall1, and NF-κB (Smith et al., 2013; Frakes et al., 2014; Buttgereit et al., 2016; Cakir et al., 2022). Additionally, current knowledge and perspectives are discussed about the use of microglia as a target for the diagnosis, monitoring and treatment of diverse pathologies.

Circadian rhythms of physiological and behavioral processes are the result of a proper communication between cells and timing cues (*Zeitgebers*; e.g., environmental light). At the molecular level, the circadian intracellular machinery consists of interlocking transcriptional-translational feedback loops (TTFL) controlled by clock genes (CG), which encode activating and inhibitory TFs [e.g., Clock, Bmal1, Pers and Crys (Hastings et al., 2018)]. These TFs operate in a time-synchronized manner via binding to specific sequences [e.g., E-box (Muñoz et al., 2002, 2006; Muñoz and Baler, 2003)], present in the regulatory regions of their target genes. These genes are known as clock-controlled genes (CCG), and they are involved in a broad range of cellular functions. In this Research Topic, Fagiani et al. review current knowledge related to the circadian molecular machinery within different brain regions and within different brain cell types, including neurons, astrocytes, and microglia (Hayashi et al., 2013; Brancaccio et al., 2017; Wang et al., 2020). The authors discuss the bidirectional interplay between the core circadian clock and various neurotransmitters [e.g., dopamine, serotonin, noradrenaline, glutamate, and GABA (Miyamoto et al., 2012; Korshunov et al., 2017; Maejima et al., 2021)], and its impact on neuronal activity and the daily timekeeping of brain functions. Discussion is included about circadian alterations in adverse contexts [e.g., AD and PD (Breen et al., 2014; Videnovic et al., 2014)]. Furthermore, they predict that a deeper understanding of the space/time/sex-dependent circadian control of brain physiology may lead to a better comprehension of circadian disruptions in the onset and progression of natural aging, as well as neurological and neurodegenerative diseases and their remedies.

Regulation (and dysregulation) by microRNAs represent a promising avenue in nervous system-related diseases, especially as biomarkers and as prognostic tools (Romano et al., 2017; Elshelmani et al., 2020, 2021). In their original article, Aggio-Bruce et al. reveal an early serum microRNA signature of retinal regression. The authors used samples from both a pre-clinical photo-oxidative damage (PD) mouse model (Natoli et al., 2016), and patients who were clinically diagnosed with either early age-related macular degeneration (AMD) (Wu et al., 2016) or late-stage AMD (Holz et al., 2017). Respective controls were used for each sample group. The expression of ~800 miRNAs was measured using OpenArray™, and differential abundance from controls was determined using the HTqPCR R package followed by pathway analysis with the DAVID functional annotation tool. The results showed that the altered circulating microRNAs correlated well with human retina pathology. Overlapping animal and human data led the authors to define a preliminary microRNA panel with higher stringency. Some of these microRNAs (e.g., let-7i/g-5p, miR-26a-5p, miR-19a-3p and miR-574-3p) may represent good candidates for early diagnosis of AMD before vision is lost. The authors also discuss similarities and discrepancies between their data and those previously published (Szemraj et al., 2015; Ren et al., 2017).

Overall, the original research and review articles of this second volume illustrate the complexity behind a healthy and diseased nervous system, and how TFs and transcription regulation are essential in pacing its development and functions. We expect this Research Topic will encourage researchers to delve deeper into the role of TFs and transcription regulation in homeostatic and adverse conditions. There is potential here to uncover novel biomarkers that could lead to new prognostic tools and new therapeutic remedies.

## Author contributions

EM: Writing—original draft, Writing—review and editing. VM: Writing—review and editing.

## References

[B1] Acampora D. Giovannantonio D. i. Simeone L. G. (2013). Otx2 is an intrinsic determinant of the embryonic stem cell state and is required for transition to a stable epiblast stem cell condition. Development 140, 43–55. 10.1242/dev.085290 23154415

[B2] Alexander T. Nolte C. Krumlauf R. (2009). Hox genes and segmentation of the hindbrain and axial skeleton. Annu. Rev. Cell Dev. Biol. 25, 431–56. 10.1146/annurev.cellbio.042308.113423 19575673

[B3] Barkai N. Shilo B. Z. (2007). Variability and robustness in biomolecular systems. Mol. Cell 28, 755–60. 10.1016/j.molcel.2007.11.013 18082601

[B4] Bellenguez C. Kucukali F. Jansen I. E. Kleineidam L. Moreno-Grau S. Amin N.. (2022). New insights into the genetic etiology of Alzheimer's disease and related dementias. Nat. Genet. 54, 412–436. 10.1038/s41588-022-01024-z 35379992 PMC9005347

[B5] Bhaduri A. Sandoval-Espinosa C. Otero-Garcia M. Oh I. Yin R. Eze U. C.. (2021). An atlas of cortical arealization identifies dynamic molecular signatures. Nature 598, 200–204. 10.1038/s41586-021-03910-8 34616070 PMC8494648

[B6] Brancaccio M. Patton A. P. Chesham J. E. Maywood E. S. Hastings M. H. (2017). Astrocytes control circadian timekeeping in the suprachiasmatic nucleus via glutamatergic signaling. Neuron 93, 1420–1435. e5. 10.1016/j.neuron.2017.02.030 28285822 PMC5376383

[B7] Breen D. P. Vuono R. Nawarathna U. Fisher K. Shneerson J. M. Reddy A. B.. (2014). Sleep and circadian rhythm regulation in early Parkinson disease. JAMA Neurol. 71, 589–595. 10.1001/jamaneurol.2014.65 24687146 PMC4119609

[B8] Broer S. Bailey C. G. Kowalczuk S. Ng C. Vanslambrouck J. M. Rodgers H.. (2008). Iminoglycinuria and hyperglycinuria are discrete human phenotypes resulting from complex mutations in proline and glycine transporters. J. Clin. Invest. 118, 3881–92. 10.1172/JCI36625 19033659 PMC2579706

[B9] Buttgereit A. Lelios I. Yu X. Vrohlings M. Krakoski N. R. Gautier E. L.. (2016). Sall1 is a transcriptional regulator defining microglia identity and function. Nat. Immunol. 17, 1397–1406. 10.1038/ni.3585 27776109

[B10] Cabal-Herrera A. M. Tassanakijpanich N. Salcedo-Arellano M. J. Hagerman R. J. (2020). Fragile X-associated tremor/ataxia syndrome (FXTAS): pathophysiology and clinical implications. Int. J. Mol. Sci. 21, 91. 10.3390/ijms21124391 32575683 PMC7352421

[B11] Cadwell C. R. Bhaduri A. Mostajo-Radji M. A. Keefe M. G. Nowakowski T. J. (2019). Development and arealization of the cerebral cortex. Neuron 103, 980–1004. 10.1016/j.neuron.2019.07.009 31557462 PMC9245854

[B12] Cakir B. Tanaka Y. Kiral F. R. Xiang Y. Dagliyan O. Wang J.. (2022). Expression of the transcription factor PU.1 induces the generation of microglia-like cells in human cortical organoids. Nat. Commun. 13, 430. 10.1038/s41467-022-28043-y 35058453 PMC8776770

[B13] Cassidy J. J. Jha A. R. Posadas D. M. Giri R. Venken K. J. Ji J.. (2013). miR-9a minimizes the phenotypic impact of genomic diversity by buffering a transcription factor. Cell 155, 1556–67. 10.1016/j.cell.2013.10.057 24360277 PMC3891883

[B14] Chapman H. Riesenberg A. Ehrman L. A. Kohli V. Nardini D. Nakafuku M.. (2018). Gsx transcription factors control neuronal versus glial specification in ventricular zone progenitors of the mouse lateral ganglionic eminence. Dev. Biol. 442, 115–126. 10.1016/j.ydbio.2018.07.005 29990475 PMC6158017

[B15] Elshelmani H. Wride M. A. Saad T. Rani S. Kelly D. J. Keegan D.. (2020). Identification of novel serum microRNAs in age-related macular degeneration. Transl. Vis. Sci. Technol. 9, 28. 10.1167/tvst.9.4.28 32818115 PMC7396178

[B16] Elshelmani H. Wride M. A. Saad T. Rani S. Kelly D. J. Keegan D.. (2021). The role of deregulated microRNAs in age-related macular degeneration pathology. Transl. Vis. Sci. Technol. 10, 12. 10.1167/tvst.10.2.12 34003896 PMC7881277

[B17] Felix M. A. Wagner A. (2008). Robustness and evolution: concepts, insights and challenges from a developmental model system. Heredity 100, 132–40. 10.1038/sj.hdy.6800915 17167519

[B18] Frakes A. E. Ferraiuolo L. Haidet-Phillips A. M. Schmelzer L. Braun L. Miranda C. J.. (2014). Microglia induce motor neuron death via the classical NF-kappaB pathway in amyotrophic lateral sclerosis. Neuron 81, 1009–1023. 10.1016/j.neuron.2014.01.013 24607225 PMC3978641

[B19] Garcia-Leon J. A. Kumar M. Boon R. Chau D. One J. Wolfs E. W. S.. (2018). SOX10 single transcription factor-based fast and efficient generation of oligodendrocytes from human pluripotent stem cells. Stem Cell Rep. 10, 655–672. 10.1016/j.stemcr.2017.12.014 29337119 PMC5830935

[B20] Guo M. Hao Y. Feng Y. Li H. Mao Y. Dong Q.. (2021). Microglial exosomes in neurodegenerative disease. Front. Mol. Neurosci. 14, 630808. 10.3389/fnmol.2021.630808 34045943 PMC8148341

[B21] Hastings M. H. Maywood E. S. Brancaccio M. (2018). Generation of circadian rhythms in the suprachiasmatic nucleus. Nat. Rev. Neurosci. 19, 453–469. 10.1038/s41583-018-0026-z 29934559

[B22] Hayashi Y. Koyanagi S. Kusunose N. Okada R. Wu Z. Tozaki-Saitoh H.. (2013). The intrinsic microglial molecular clock controls synaptic strength via the circadian expression of cathepsin S. Sci. Rep. 3, 2744. 10.1038/srep02744 24067868 PMC3783043

[B23] Hazrati A. Soudi S. Malekpour K. Mahmoudi M. Rahimi A. Hashemi S. M.. (2022). Immune cells-derived exosomes function as a double-edged sword: role in disease progression and their therapeutic applications. Biomark Res. 10, 30. 10.1186/s40364-022-00374-4 35550636 PMC9102350

[B24] Hernandez-Miranda L. R. Birchmeier C. (2015). CO(2) in the spotlight. Elife 4, 86. 10.7554/eLife.08086 25970131 PMC4429524

[B25] Hernandez-Miranda L. R. Ibrahim D. M. Ruffault P. L. Larrosa M. Balueva K. Muller T.. (2018). Mutation in LBX1/Lbx1 precludes transcription factor cooperativity and causes congenital hypoventilation in humans and mice. Proc. Natl. Acad. Sci. USA. 115, 13021–13026. 10.1073/pnas.1813520115 30487221 PMC6304989

[B26] Hernandez-Miranda L. R. Ruffault P. L. Bouvier J. C. Murray A. J. Morin-Surun M. P. Zampieri N.. (2017). Genetic identification of a hindbrain nucleus essential for innate vocalization. Proc. Natl. Acad. Sci. USA. 114, 8095–8100. 10.1073/pnas.1702893114 28698373 PMC5544295

[B27] Heydari T. M, A. L. Fisher C. L. Aguilar-Hidalgo D. Shukla S. Yachie-Kinoshita A. Hughes M.. (2022). IQCELL: a platform for predicting the effect of gene perturbations on developmental trajectories using single-cell RNA-seq data. PLoS Comput. Biol. 18, e1009907. 10.1371/journal.pcbi.1009907 35213533 PMC8906617

[B28] Holz F. G. Sadda S. R. Staurenghi G. Lindner M. Bird A. C. Blodi B. A.. (2017). Imaging protocols in clinical studies in advanced age-related macular degeneration: recommendations from classification of atrophy consensus meetings. Ophthalmology 124, 464–478. 10.1016/j.ophtha.2016.12.002 28109563

[B29] Isik E. G. Hernandez-Miranda L. R. (2022). Early development of the breathing network. Handb. Clin. Neurol. 188, 125–149. 10.1016/B978-0-323-91534-2.00002-3 35965024

[B30] Kalluri R. Lebleu V. S. (2020). The biology, function, and biomedical applications of exosomes. Science 367, 6977. 10.1126/science.aau6977 32029601 PMC7717626

[B31] Kaltschmidt B. Kaltschmidt C. (2009). NF-kappaB in the nervous system. Cold Spring Harb. Perspect. Biol. 1, a001271. 10.1101/cshperspect.a001271 20066105 PMC2773634

[B32] Kaltschmidt C. Greiner J. F. W. Kaltschmidt B. (2021). The transcription factor NF-kappaB in stem cells and development. Cells 10, 2024. 10.3390/cells10082042 34440811 PMC8391683

[B33] Kandel E. R. (2001). The molecular biology of memory storage: a dialogue between genes and synapses. Science 294, 1030–8. 10.1126/science.1067020 11691980

[B34] Korshunov K. S. Blakemore L. J. Trombley P. Q. (2017). Dopamine: A Modulator of Circadian Rhythms in the Central Nervous System. Front. Cell. Neurosci., 11, 91. 10.3389/fncel.2017.00091 28420965 PMC5376559

[B35] Lange M. Bergen V. Klein M. Setty M. Reuter B. Bakhti M. Pe'er D.. (2022). CellRank for directed single-cell fate mapping. Nat. Methods 19, 159–170. 10.1038/s41592-021-01346-6 35027767 PMC8828480

[B36] Lee H. Nowosiad P. Dutan Polit L. M. Price J. Srivastava D. P. Thuret S.. (2020). Apolipoprotein E expression pattern in human induced pluripotent stem cells during *in vitro* neural induction. F1000Res 9, 353. 10.12688/f1000research.23580.1 32685135 PMC7355222

[B37] Lowenstein E. D. Cui K. Hernandez-Miranda L. R. (2023). Regulation of early cerebellar development. FEBS J. 290, 2786–2804. 10.1111/febs.16426 35262281

[B38] Mader S. Brimberg L. (2019). Aquaporin-4 water channel in the brain and its implication for health and disease. Cells 8, 90. 10.3390/cells8020090 30691235 PMC6406241

[B39] Maejima T. Tsuno Y. Miyazaki S. Tsuneoka Y. Hasegawa E. Islam M. T.. (2021). GABA from vasopressin neurons regulates the time at which suprachiasmatic nucleus molecular clocks enable circadian behavior. Proc. Natl. Acad. Sci. USA. 3, 118. 10.1073/pnas.2010168118 33526663 PMC8017960

[B40] Miquelajauregui A. Van De Putte T. Polyakov A. Nityanandam A. Boppana S. Seuntjens E.. (2007). Smad-interacting protein-1 (Zfhx1b) acts upstream of Wnt signaling in the mouse hippocampus and controls its formation. Proc. Natl. Acad. Sci. USA. 104, 12919–24. 10.1073/pnas.0609863104 17644613 PMC1929013

[B41] Miyamoto H. Nakamaru-Ogiso E. Hamada K. Hensch T. K. (2012). Serotonergic integration of circadian clock and ultradian sleep-wake cycles. J. Neurosci. 32, 14794–803. 10.1523/JNEUROSCI.0793-12.2012 23077063 PMC6621427

[B42] Mowat D. R. Croaker G. D. Cass D. T. Kerr B. A. Chaitow J. Ades L. C.. (1998). Hirschsprung disease, microcephaly, mental retardation, and characteristic facial features: delineation of a new syndrome and identification of a locus at chromosome 2q22-q23. J. Med. Genet. 35, 617–23. 10.1136/jmg.35.8.617 9719364 PMC1051383

[B43] Muñoz E. Baler R. (2003). The circadian E-box: when perfect is not good enough. Chronobiol. Int. 20, 371–88. 10.1081/CBI-120022525 12868535

[B44] Muñoz E. Brewer M. Baler R. (2002). Circadian transcription. Thinking outside the E-Box. J. Biol. Chem. 277, 36009–17. 10.1074/jbc.M203909200 12130638

[B45] Muñoz E. Brewer M. Baler R. (2006). Modulation of BMAL/CLOCK/E-Box complex activity by a CT-rich cis-acting element. Mol. Cell. Endocrinol. 252, 74–81. 10.1016/j.mce.2006.03.007 16650525

[B46] Muñoz E. M. (2018). Microglia-precursor cell interactions in health and in pathology. Biocell 42, 41–45. 10.32604/biocell.2018.07011

[B47] Muñoz E. M. (2022). Microglia in circumventricular organs: the pineal gland example. ASN Neuro. 14, 17590914221135697. 10.1177/17590914221135697 36317305 PMC9629557

[B48] Muñoz E. M. Martinez Cerdeño V. De Souza F. S. J. Rath M. F. eds. (2022). Transcription regulation—Brain Development and Homeostasis—A finely tuned and orchestrated scenario in physiology and pathology. Front. Media SA. 4, 2. 10.3389./978-2-88974-457-2 35095419 PMC8795980

[B49] Muñoz E. M. Souza D. e. Rath F. S. J. M. F. Martinez Cerdeño V. (2021). Editorial: transcription regulation-brain development and homeostasis-a finely tuned and orchestrated scenario in physiology and pathology. Front. Mol. Neurosci. 14, 834607. 10.3389/fnmol.2021.834607 35095419 PMC8795980

[B50] Natoli R. Jiao H. Barnett N. L. Fernando N. Valter K. Provis J. M.. (2016). A model of progressive photo-oxidative degeneration and inflammation in the pigmented C57BL/6J mouse retina. Exp. Eye Res. 147, 114–127. 10.1016/j.exer.2016.04.015 27155143

[B51] Panicker N. Ge P. Dawson V. L. Dawson T. M. (2021). The cell biology of Parkinson's disease. J. Cell Biol. 220, 95. 10.1083/jcb.202012095 33749710 PMC8103423

[B52] Rath M. F. Muñoz E. Ganguly S. Morin F. Shi Q. Klein D. C.. (2006). Expression of the Otx2 homeobox gene in the developing mammalian brain: embryonic and adult expression in the pineal gland. J. Neurochem. 97, 556–66. 10.1111/j.1471-4159.2006.03773.x 16539656

[B53] Ren C. Liu Q. Wei Q. Cai W. He M. Du Y.. (2017). Circulating miRNAs as potential biomarkers of age-related macular degeneration. Cell. Physiol. Biochem. 41, 1413–1423. 10.1159/000467941 28315863

[B54] Roesch S. Rasp G. Sarikas A. Dossena S. (2021). Genetic determinants of non-syndromic enlarged vestibular aqueduct: a review. Audiol. Res. 11, 423–442. 10.3390/audiolres11030040 34562878 PMC8482117

[B55] Romano G. L. Platania C. B. M. Drago F. Salomone S. Ragusa M. Barbagallo C. A.. (2017). Retinal and circulating miRNAs in age-related macular degeneration: an *in vivo* animal and human study. Front. Pharmacol. 8, 168. 10.3389/fphar.2017.00168 28424619 PMC5371655

[B56] Sagner A. Briscoe J. (2017). Morphogen interpretation: concentration, time, competence, and signaling dynamics. Wiley Interdiscip. Rev. Dev. Biol. 6, 271. 10.1002/wdev.271 28319331 PMC5516147

[B57] Schuster J. Sobol M. Fatima A. Khalfallah A. Laan L. Anderlid B. M.. (2019). Mowat-Wilson syndrome: generation of two human iPS cell lines (UUIGPi004A and UUIGPi005A) from siblings with a truncating ZEB2 gene variant. Stem Cell Res. 39, 101518. 10.1016/j.scr.2019.101518 31376723

[B58] Shi Y. Kirwan P. Livesey F. J. (2012). Directed differentiation of human pluripotent stem cells to cerebral cortex neurons and neural networks. Nat. Protoc. 7, 1836–46. 10.1038/nprot.2012.116 22976355

[B59] Sierra A. Castro De. Del Rio-Hortega F. Rafael Iglesias-Rozas J. Garrosa J. M. Kettenmann H. (2016). The “Big-Bang” for modern glial biology: translation and comments on Pio del Rio-Hortega 1,919 series of papers on microglia. Glia 64, 1801–40. 10.1002/glia.23046 27634048

[B60] Silbereis J. C. Pochareddy S. Zhu Y. Li M. Sestan N. (2016). The cellular and molecular landscapes of the developing human central nervous system. Neuron 89, 248–68. 10.1016/j.neuron.2015.12.008 26796689 PMC4959909

[B61] Smith A. M. Gibbons H. M. Oldfield R. L. Bergin P. M. Mee E. W. Faull R. L.. (2013). The transcription factor PU.1 is critical for viability and function of human brain microglia. Glia 61, 929–42. 10.1002/glia.22486 23483680

[B62] Smith D. Shimamura T. Barbera S. Bejcek B. E. (2008). NF-kappaB controls growth of glioblastomas/astrocytomas. Mol. Cell. Biochem. 307, 141–7. 10.1007/s11010-007-9593-4 17828582

[B63] Snow W. M. Albensi B. C. (2016). Neuronal gene targets of NF-kappaB and their dysregulation in Alzheimer's disease. Front. Mol. Neurosci. 9, 118. 10.3389/fnmol.2016.00118 27881951 PMC5101203

[B64] Stolt C. C. Schmitt S. Lommes P. Sock E. Wegner M. (2005). Impact of transcription factor Sox8 on oligodendrocyte specification in the mouse embryonic spinal cord. Dev. Biol. 281, 309–17. 10.1016/j.ydbio.2005.03.010 15893981

[B65] Swarna M. Jyothy A. Usha Rani P. Reddy P. P. (2004). Amino acid disorders in mental retardation: a two-decade study from Andhra Pradesh. Biochem. Genet. 42, 85–98. 10.1023/B:BIGI.0000020464.05335.79 15168722

[B66] Szczygielski J. Kopanska M. Wysocka A. Oertel J. (2021). Cerebral microcirculation, perivascular unit, and glymphatic system: role of aquaporin-4 as the gatekeeper for water homeostasis. Front. Neurol. 12, 767470. 10.3389/fneur.2021.767470 34966347 PMC8710539

[B67] Szemraj M. Bielecka-Kowalska A. Oszajca K. Krajewska M. Gos R. Jurowski P.. (2015). Serum microRNAs as potential biomarkers of AMD. Med. Sci. Monit. 21, 2734–42. 10.12659/MSM.893697 26366973 PMC4576928

[B68] Takanaga H. Mackenzie B. Suzuki Y. Hediger M. A. (2005). Identification of mammalian proline transporter SIT1 (SLC6A20) with characteristics of classical system imino. J. Biol. Chem. 280, 8974–84. 10.1074/jbc.M413027200 15632147

[B69] Van Den Berghe V. Stappers E. Vandesande B. Dimidschstein J. Kroes R. Francis A.. (2013). Directed migration of cortical interneurons depends on the cell-autonomous action of Sip1. Neuron 77, 70–82. 10.1016/j.neuron.2012.11.009 23312517

[B70] Videnovic A. Lazar A. S. Barker R. A. Overeem S. (2014). 'The clocks that time us'–circadian rhythms in neurodegenerative disorders. Nat. Rev. Neurol. 10, 683–93. 10.1038/nrneurol.2014.206 25385339 PMC4344830

[B71] Wang X. L. Wolff S. E. C. Korpel N. Milanova I. Sandu C. Rensen P. C. N.. (2020). Deficiency of the circadian clock Gene Bmal1 reduces microglial immunometabolism. Front. Immunol. 11, 586399. 10.3389/fimmu.2020.586399 33363534 PMC7753637

[B72] Wang Y. Yi K. Liu X. Tan Y. Jin W. Li Y.. (2021). HOTAIR up-regulation activates NF-kappaB to induce immunoescape in gliomas. Front. Immunol. 12, 785463. 10.3389/fimmu.2021.785463 34887871 PMC8649724

[B73] Wolf F. A. Hamey F. K. Plass M. Solana J. Dahlin J. S. Gottgens B.. (2019). PAGA: graph abstraction reconciles clustering with trajectory inference through a topology preserving map of single cells. Genome Biol. 20, 59. 10.1186/s13059-019-1663-x 30890159 PMC6425583

[B74] Wu Z. Ayton L. N. Luu C. D. Baird P. N. Guymer R. H. (2016). Reticular pseudodrusen in intermediate age-related macular degeneration: prevalence, detection, clinical, environmental, and genetic associations. Invest. Ophthalmol. Vis. Sci. 57, 1310–6. 10.1167/iovs.15-18682 26998717

[B75] Zhong S. Zhang S. Fan X. Wu Q. Yan L. Dong J.. (2018). A single-cell RNA-seq survey of the developmental landscape of the human prefrontal cortex. Nature 555, 524–528. 10.1038/nature25980 29539641

[B76] Zweier C. Albrecht B. Mitulla B. Behrens R. Beese M. Gillessen-Kaesbach G.. (2002). “Mowat-Wilson” syndrome with and without Hirschsprung disease is a distinct, recognizable multiple congenital anomalies-mental retardation syndrome caused by mutations in the zinc finger homeo box 1B gene. Am. J. Med. Genet. 108, 177–81. 10.1002/ajmg.10226 11891681

